# The Tensile Properties, Scratch Behaviors and Sliding Wear of Oxide Scale Formed on Titanium Grade 2

**DOI:** 10.3390/ma13143048

**Published:** 2020-07-08

**Authors:** Krzysztof Aniołek, Adrian Barylski, Marian Kupka, Iwona Leszek

**Affiliations:** Institute of Materials Engineering, University of Silesia, ul. 75 Pułku Piechoty 1A, 41-500 Chorzów, Poland; adrian.barylski@us.edu.pl (A.B.); marian.kupka@us.edu.pl (M.K.); iwona.lubach@gmail.com (I.L.)

**Keywords:** titanium, isothermal oxidation, oxide layers, tensile properties, tribological properties, adhesion

## Abstract

The tensile properties, scratch behaviors and sliding wear of an oxide scale obtained on the surface of titanium Grade 2 in the process of isothermal oxidation at 600, 700 and 800 °C were determined in the study. It was shown that the intensity of the oxidation process increased along with an increase in temperature and extension of the oxidation time, which translated directly into the thickness of the deposited oxide layers. The tests showed that isothermal oxidation had an adverse effect on the tensile properties of titanium. After oxidation, it was found that the maximum reduction in tensile strength, R_m_, was approximately 17.5%, and of the yield point, R_p0.2_, approximately 13.9%. Examination of scratch behaviors of the oxide scale showed that the layers obtained at temperatures of 700 (72 h) and 800 °C (2 and 6 h) had the best adhesion properties. The best resistance to scratching was exhibited by the layer obtained after 6 h oxidation at 800 °C (critical load: L_c1_ = 63 N, L_c2_ = 85 N). The study showed that after oxidation, a considerable reduction in wear factor of a disc made of titanium Grade 2 was observed for both the friction couples used (Al_2_O_3_, steel 100Cr6). The maximum reduction in wear factor of the oxidized titanium disc during interaction with Al_2_O_3_ balls was ca. 79%, and with 100Cr6 balls, ca. 96%.

## 1. Introduction

The dynamic development of technical and biomedical engineering has become a driving force for the search for new or improvement of existing materials which will be able to meet the ever-increasing demand for better performance properties. This demand necessitates the use of increasingly advanced engineering and biomaterials with more favorable mechanical and fatigue characteristics, as well as an increased resistance to corrosion and wear in friction conditions.

Titanium and its alloys, due to their very favorable performance characteristics, belong to the materials which are often used in industrial areas and in the biomedical sector. The popularity of these materials results from their high yield strength to tensile strength ratio (R_p0.2_/R_m_), low density, excellent corrosion resistance and the best, compared with other metallic biomaterials, biocompatibility [1,2,3,4,5,6]. However, despite their unquestionable advantages, these materials have poor tribological properties, which limits their use in friction couples [7,8]. The main problem in technical applications of titanium and its alloys is a high coefficient of friction and a tendency to adhesive wear, which leads to failures at an early stage of operation [9,10,11].

In recent years, a number of surface treatment techniques have been used to improve the frictional characteristics of these materials [12,13,14]. One of the most effective methods to increase the resistance to sliding wear is isothermal oxidation, which, as demonstrated in the literature, may be more effective in improving the abrasion resistance of titanium and its alloys than ion nitriding [15]. The high quality of oxide scales obtained by isothermal oxidation is primarily a function of the oxidation process parameters.

The essential requirement for coatings and layers is the appropriate adhesion to the surface in which they are embedded [16,17]. The prerequisite for good adhesion of coatings to the substrate is the very high cleanliness of the substrate before the application of a coating. Apart from the cleanliness of the substrate surface, the following factors affect the adhesion of the coating: internal stresses, the degree of the substrate surface development (the greater the roughness, the better adhesion) and the difference in plasticity between the substrate material and the coating [17]. Good adhesion of coatings prevents them from peeling off and spalling as a result of temperature or strong external activity of mostly mechanical nature.

The adhesion and tribological properties of oxides produced on titanium materials depend, first of all, on the oxidation treatment parameters. As the temperature increases, layers of higher thickness, but more susceptible to scale spalling, are obtained [18]. It is an extremely important challenge to find the optimal temperature and time conditions which will allow obtaining oxide scales with very good performance and adhesion properties. Expansion of oxide layers, as well as the interaction of other processes at the metal/scale boundary are the cause of the loss of good adhesion to the substrate. Analysis of the literature has shown a considerable gap in the area of tensile, tribological and adhesive characteristics of oxide scales obtained on titanium. There is missing information in the literature on the influence of temperature and time on the adhesion of oxide scales and tensile properties of titanium after high-temperature oxidation. The friction characteristics of titanium Grade 2 after annealing, depending on the assumed oxidation conditions, also need to be complemented.

In this study, pure titanium was subjected to isothermal oxidation. For the first time, the scratch behavior and tensile properties of oxide scales on titanium were determined after high-temperature oxidation. In addition, as part of the study, the thickness and wear behavior of the oxide scales obtained on titanium after interaction with ceramic balls (Al_2_O_3_) and steel balls (bearing steel 100Cr6) were determined.

## 2. Experimental Procedures

Titanium Grade 2 rods with diameters of 12 (examination of oxide layers’ thickness), 20 (scratch test) and 40 mm (tribological tests) were used in the tests. Specimens for the measurement of thickness and adhesion of the oxide layers were ground using abrasive paper with a grit size of 300, 600, 1200, 2000 and 2500, polished with diamond pastes and cleaned in acetone. Specimens for tribological tests were ground with abrasive paper of 300, 600, 800 and 1200 grit size, and then cleaned in acetone. Oxidation of the specimens was conducted in a resistance furnace (Czylok, Jastrzębie-Zdrój, Poland) in an air atmosphere. In order to vary the thickness and properties of the oxide scale, the annealing process was carried out at temperatures of 600 and 700 °C over a period of 6, 24, 48 and 72 h, and at 800 °C over a period of 2 and 6 h (the time of oxidation at 800 °C was shortened due to the formation of an oxide scale characterized by poor adhesion). Based on the microscopic observations and thickness measurements of the formed oxide scale, it was decided that further studies would be carried out for the following oxidation variants: 600 °C/24 h, 600 °C/72 h, 700 °C/24 h, 700 °C/72 h, 800 °C/2 h and 800 °C/6 h.

Measurements of oxide thicknesses were performed using a JSM 6480 (Jeol, Tokio, Japan) scanning electron microscope. The microscope software (SEM Control User Interface) and photographs taken at 5000× magnification were used to this end. Several microscopic photographs were used for each test variant, with 10 measurements taken on each photo. The paper presents the averaged results.

Tests aiming at the determination of the effect of isothermal oxidation on the tensile properties of titanium Grade 2 were performed using an INSTRON 5982 machine (Instron, USA). A video extensometer was used to accurately measure the specimen strain during the test. Tests were performed for the as-received material and for the material after annealing at temperatures of 600, 700 (24 h, 72 h) and 800 °C (2 h, 6 h). Strength tests were carried out at ambient temperature. Specimens with a length l_0_ = 25 mm and a diameter d_0_ = 5 mm were used in the tests. During the test, the beam displaced at a rate of 1 mm/min. The tensile strength R_m_, yield point R_p0.2_, elongation A and reduction in area Z were determined in the tests.

Scratch behaviors of the layers formed on pure titanium were examined using a Revetest Xpress tester (CSM, Corcelles-Cormondrèche, Switzerland), by means of the scratch test method. The tests were performed in accordance with the ASTM_C1624 standard. Each test was performed at three stages. In the first stage, the specimen profile was scanned under a load of 1 N. In the next stage, the load of the indenter increased from 1 to 100 N. In the last stage of the test, the profile of the scratch formed in the second (scan) stage was scanned. The indenter movement rate was 7.6 mm/min. The length of the scratch formed during the tests was 15 mm. The following parameters were determined in the tests:

- F_n_—indenter load;
- F_t_—resistance to indenter penetration;
- P_d_—depth of indenter penetration under load;
- R_d_—scratch depth after unloading the indenter.

Analysis of the parameters obtained in the scratch test allowed the determination of critical force values, L_c1_ (first damage to oxide scale) and L_c2_ (detachment of oxide scale from the substrate).

Dry sliding wear tests were performed on an TRN tribometer (Anton Paar, Corcelles-Cormondrèche, Switzerland). Tribological tests were performed on titanium specimens with a diameter of 40 mm (the height of disc specimens was 5 mm), with 6 mm diameter ceramic balls made of Al_2_O_3_ and high-carbon steel (100Cr6) acting as counter-specimens. For the research, Al_2_O_3_ balls were used, since this material is often applied in medicine, as are titanium and its alloys. Moreover, aluminum oxide (Al_2_O_3_) is a widely used material for abrasion resistance testing of titanium and its alloys. In turn, bearing steel 100Cr6 was chosen to compare tribological characteristics with the basic ceramic couple: titanium-Al_2_O_3_. Steel 100Cr6 is also a standard material used and recommended as a counter-specimen in many tribological testers. The tests were conducted for the following parameters: load—5 N, linear speed—0.1 m/s, length of the path of friction—1000 m. For each test variant, 4 repetitions were done. The wear factor was determined for the disc made of titanium Grade 2, as well as for the Al_2_O_3_ and 100Cr6 balls, and calculated using the formula

(1) $$V_{v}=\frac{V}{F\times s}$$

where:

- *V_v_*—wear factor (mm^3^/N·m);
- *V*—volume of the material removed during tests (mm^3^);
- *F*—load (N);
- *s*—length of the path of friction (m).

The volume of the material of Al_2_O_3_ and 100Cr6 balls removed as a result of friction was determined using a SJ500 profilometer (Mitutoyo, Tokio, Japan). For the calculation of wear factor of the balls (1), the following formula was applied:

(2) $$V=\pi \frac{b^{4}}{64\times R}$$

where:

- *V*—volume of the material removed (mm^3^);
- *b*—diameter of the wear track of the ball, measured in two perpendicular directions (mm);
- *R*—diameter of the ball (mm).

For surface roughness measurements after oxidation, a Mitutoyo SJ500 contact profilometer was used. The tests were carried out over a measuring length of 5 mm. In order to obtain reliable and repeatable test results, 5 measurements were taken on each sample. In the measurements of the geometrical structure, the following parameters were determined: R_a_—mean arithmetic deviation of profile ordinates, R_z_—the greatest height of a profile, R_q_—mean square deviation of a profile, R_p_—height of the highest profile elevation.

## 3. Results and Discussion

### 3.1. Oxide Scale Thickness

**Figure 1:**
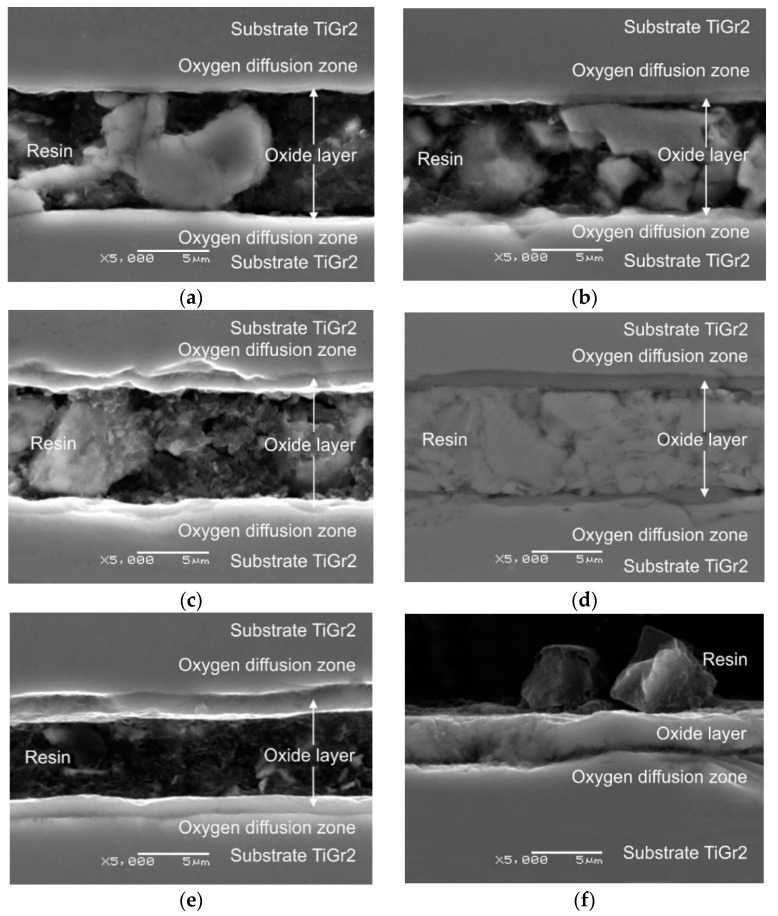

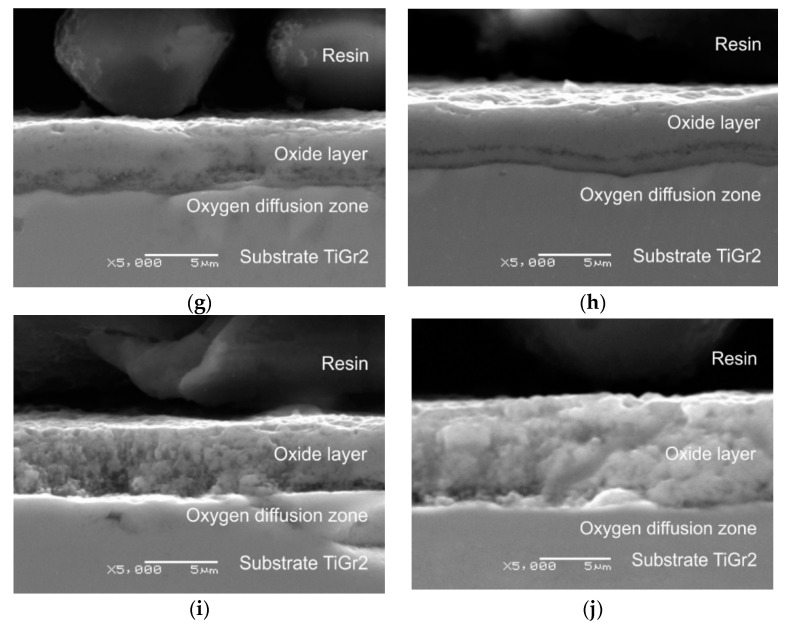
Cross-sectional area of titanium with an oxide scale obtained at a temperature of: 600 °C for (**a**) 6 h, (**b**) 24 h, (**c**) 48 h and (**d**) 72 h; (**e**) 700 °C for 6 h, (**f**) 24 h, (**g**) 48 h and (**h**) 72 h; (**i**) 800 °C for 2 h and (**j**) 6 h.

**Figure 2:**
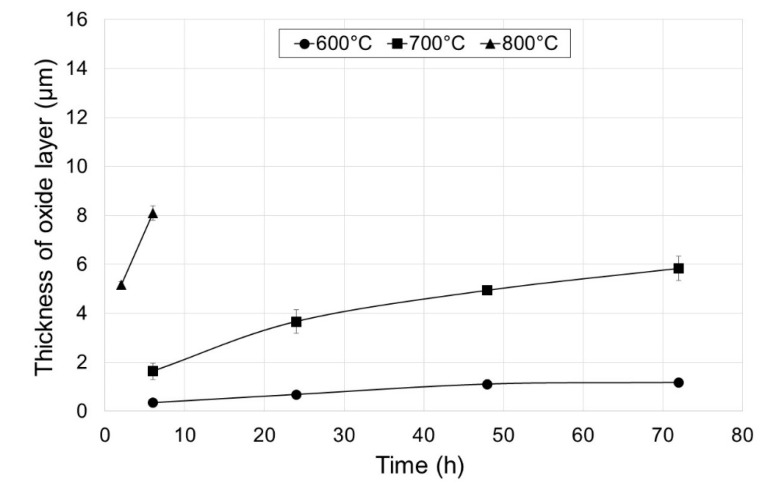
Results of thickness measurements of oxide scales depending on annealing parameters.

Figure 1a–h shows photographs of the oxide scale obtained after the annealing of titanium Grade 2 at 600 and 700 °C for 6, 24, 48 and 72 h. Figure 1i–j presents microscopic images of the layers produced at 800 °C over a period of 2 and 6 h (the oxidation process was shortened due to its high intensity, thereby allowing to avoid the tendency of oxide scales to flaking and spalling). Figure 2 presents a graph with thicknesses of the layers formed, depending on the annealing parameters.

Microscopic observations showed that the oxide scale formed on the titanium surface at 600 and 700 °C was of good quality and characterized by a homogeneous structure and thickness (Figure 1). In addition, it was found that the oxidation parameters (both time and temperature) had a significant influence on the thickness of the oxides formed. Previous studies by the authors [19] showed that the oxidation process was consistent with a parabolic curve and its intensity increased primarily as the oxidation temperature increased. In paper [20], it was affirmed that in such conditions, passive oxidation takes place on the surface (the oxides formed tended to remain linked on the surface and did not evaporate).

The oxide scales obtained after annealing at 600 °C had thicknesses from 0.35 to 1.18 µm. Similar results were achieved in study [21] after titanium oxidation at a temperature of 625 °C. It was shown in the study that extension of the annealing time resulted in the formation of more than three times thicker oxide scales. As results from paper [22], extending the oxidation time allows obtaining oxides with better functional properties. However, Dalili et al. [23] state that too long oxidation may lead to obtaining oxides prone to delamination. A change in the annealing temperature from 600 to 700 °C led to a considerable increase in the oxide scale thickness. After thermal treatment at 700 °C, the oxide layers had a thickness from 1.63 to 5.83 µm. At the same time, after oxidation at 800 °C for 2 h, the thickness of the oxide scale formed (ca. 5.5 µm) was similar to the layer obtained at 700 °C over a period of 72 h. After annealing at 800 °C, the obtained oxide thickness was the highest (ca. 8 µm). Examination results of the phase composition of the obtained oxides were presented in our previous paper [19]. XRD (X-ray Diffraction) examinations showed that oxide scales were mostly composed of TiO_2_ in its rutile form. In addition, presence of the Ti_3_O phase was found after annealing at temperatures of 600 and 700 °C. No other oxides were found. At the same time, it was shown that shortening the oxidation time allowed obtaining good-quality oxide scales despite the high intensity of the diffusion processes at 800 °C. It was demonstrated in the studies that the scale formed on titanium Grade 2 was considerably thicker compared with the similar oxidation parameters of the titanium Ti-6Al-7Nb alloy [24]. A lower thickness of oxide layers on titanium alloys is related to the presence of alloying additions, e.g., Nb, which often inhibit the oxidation process [25].

### 3.2. Tensile Properties of Titanium after Annealing

**Figure 3:**
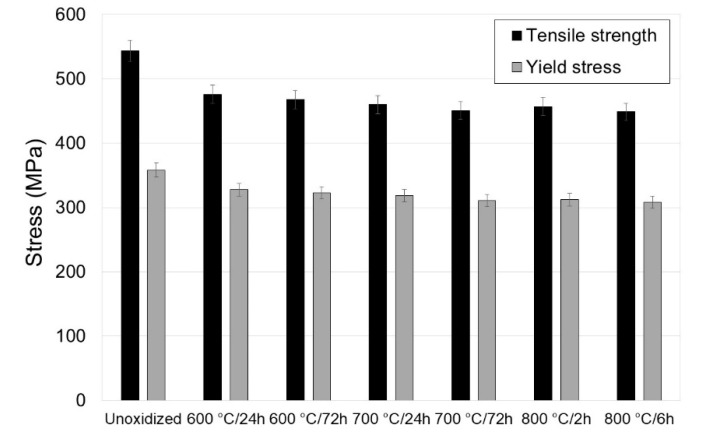
Tensile properties of titanium after annealing.

**Figure 4:**
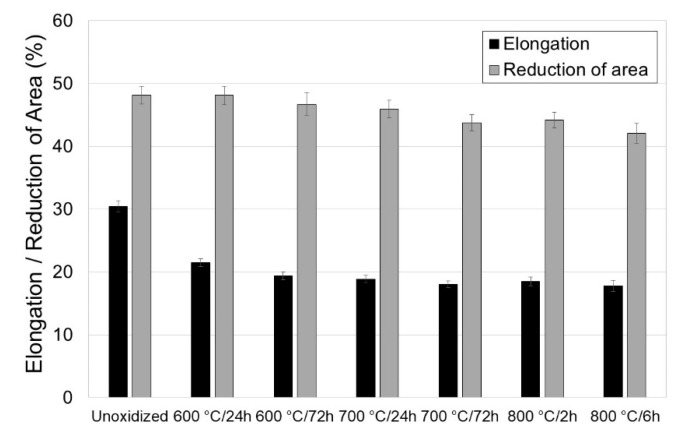
Measurement results of plastic parameters (elongation and reduction in area) for titanium Grade 2.

**Figure 5:**
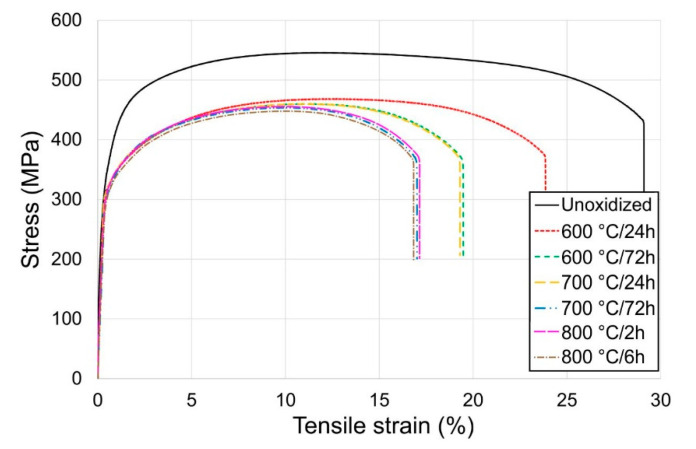
Examples of tensile curves of titanium before and after annealing.

Figure 3, Figure 4 and Figure 5 present the tensile properties before and after modification of the titanium surface at temperatures of 600, 700 (24 h, 72 h) and 800 °C (2 h, 6 h).

It was shown in the tests that titanium Grade 2 in the non-oxidized condition had the best mechanical and plastic properties. Isothermal oxidation was found to adversely affect the tensile properties of titanium Grade 2. It was shown that the principal parameters determined in the tensile test, such as: tensile strength (R_m_), yield point (R_p0.2_), elongation (A) and reduction in area (Z), decreased with the temperature increase and extension of the oxidation time (Figure 3 and Figure 4). After isothermal oxidation, it was found that the maximum reduction in tensile strength was 95 MPa (reduction by ca. 17.5%), and in yield point, 49.7 MPa (reduction by ca. 13.9%). At the same time, the maximum reduction in elongation was ca. 12.7%, and the reduction in area was ca. 6.1%. Thus, it was found that isothermal oxidation to a larger degree negatively affected the elongation parameter. The deterioration of the mechanical properties of titanium after isothermal oxidation may have been caused by the oxidation of grain boundaries and thereby cracking along the boundaries [26].

### 3.3. Examination of Adhesive Properties by the Scratch Test Method

**Figure 6:**
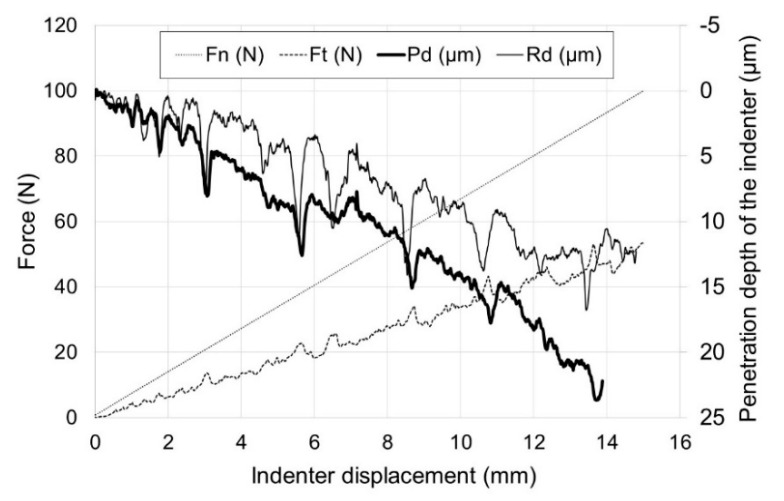
Scratch behavior of titanium Grade 2 in the non-oxidized condition (F_n_—indenter load, F_t_—resistance to indenter penetration, P_d_—depth of indenter penetration under load, R_d_—depth of indenter penetration after unloading).

**Figure 7:**
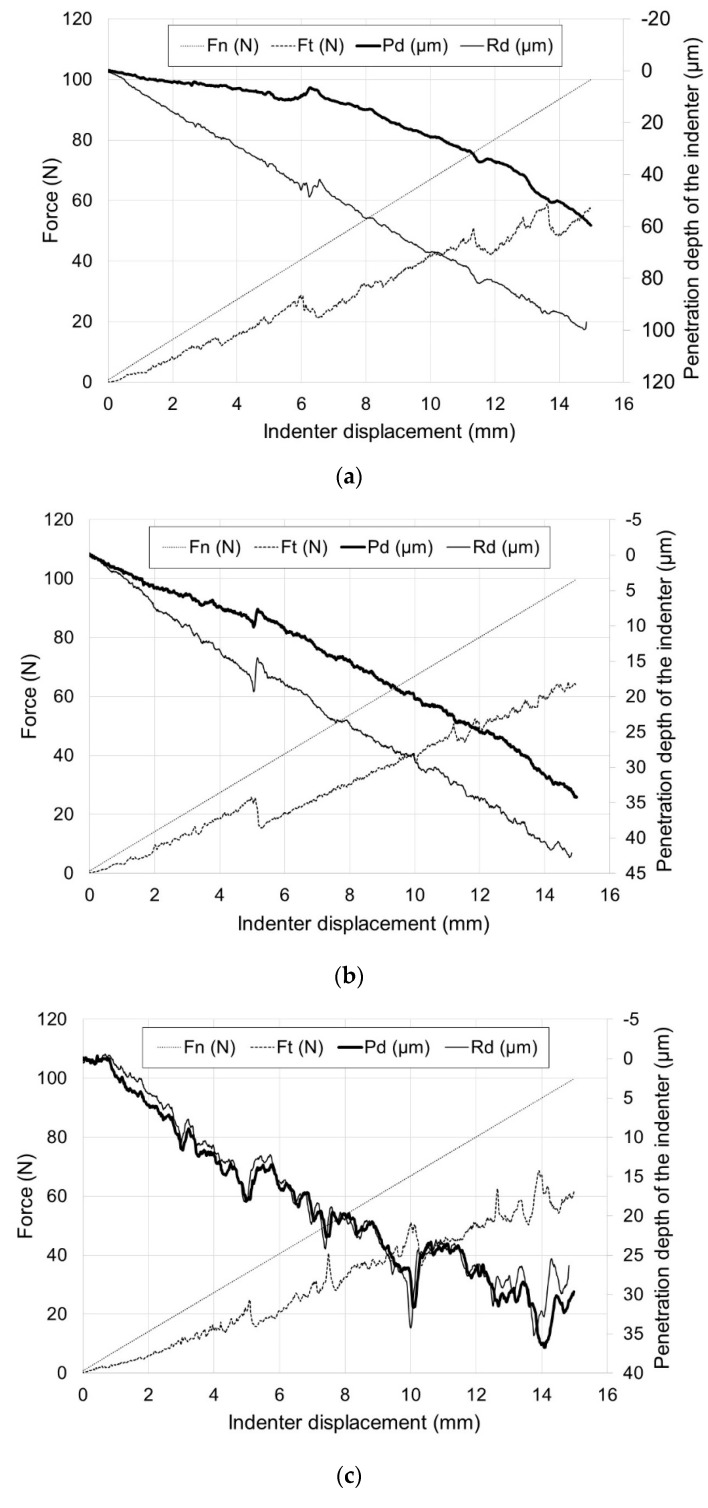

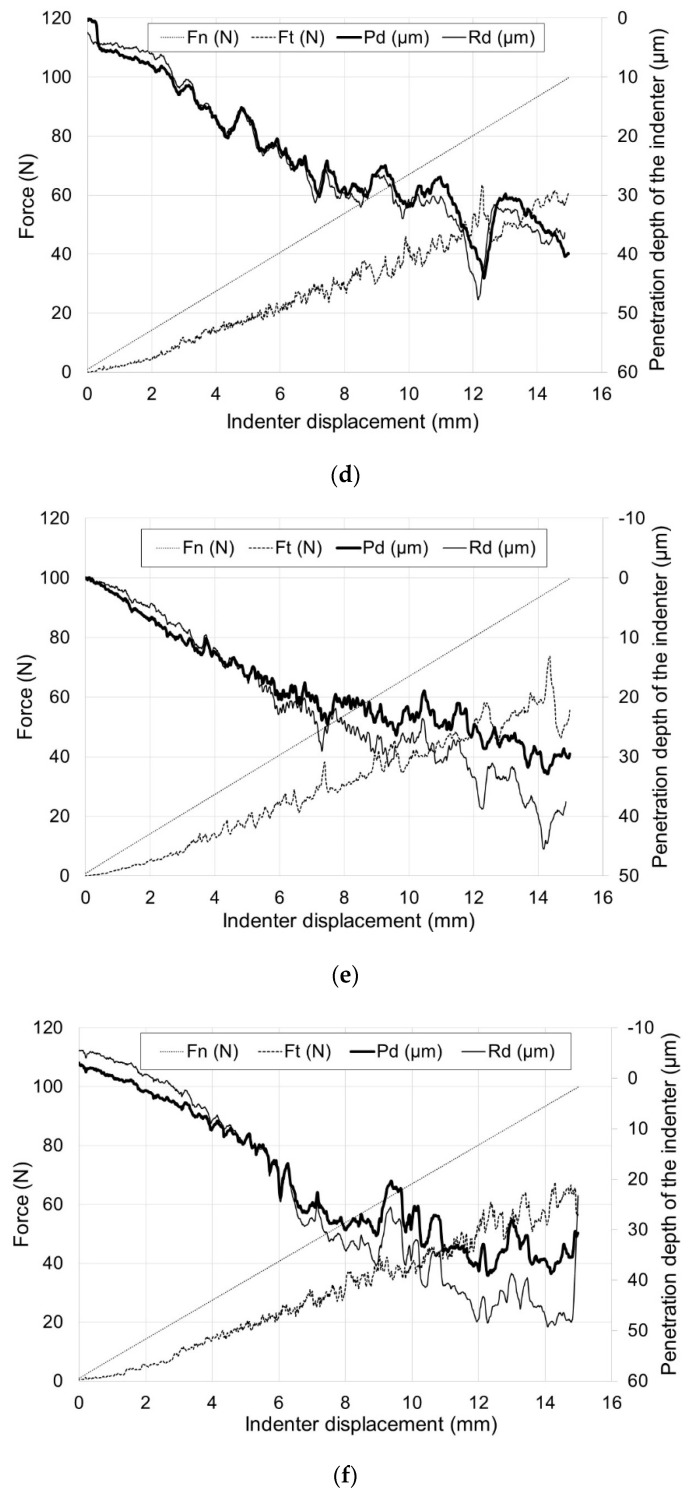
Scratch behavior of titanium after annealing at: (**a**) 600 °C for 24 h, (**b**) 600 °C for 72 h, (**c**) 700 °C for 24 h, (**d**) 700 °C for 72 h, (**e**) 800 °C for 2 h, (**f**) 800 °C for 6 h (F_n_—indenter load, F_t_—resistance to indenter penetration, P_d_—depth of indenter penetration under load, R_d_—depth of indenter penetration after unloading).

Figure 6 shows the results of a scratch test for pure titanium. Figure 7 presents the scratch behaviors of oxide scales obtained on titanium. Table 1 presents the critical load values, L_c1_ and L_c2_, determined on the basis of the parameters recorded in the scratch test. Analysis of the parameters (F_n_, F_t_, P_d_, R_d_) for the non-oxidized specimen showed the absence of damage throughout the scratch length and its stable course. As can be seen from the obtained graph, the increase in the resistance force during the movement of the indenter (F_t_) was in its nature similar to linear.

The layer obtained on titanium was characterized by good adhesion properties. The oxide scales with insignificant thicknesses, produced at 600 °C, showed the first damage at a critical load L_c1_ of ca. 35–39 N. The layer got completely torn off from the substrate at load L_c2_ of 75 N. Similar parameters of critical loads were obtained for the layer which formed for 24 h at 700 °C. The oxide scale obtained at a temperature of 700 (72 h) and 800 °C (2 and 6 h) exhibited the best scratch resistance. The first damage to the oxide scale was observed at loads of: 47 N (700 °C/72 h), 50 N (800 °C/2 h) and 63 N (800 °C/6 h), respectively. The value of critical load L_c2_ for the above oxidation variants was over 80 N. The best adhesion properties were exhibited by the layer after annealing at 800 °C for 6 h (L_c1_ = 63 N, L_c2_ = 85 N). Compared with the similar oxidation parameters of Ti-6Al-7Nb alloy discussed in paper [18], the layer obtained on titanium showed a higher scratch resistance, which may be connected with the greater intensity of the diffusion processes which take place during titanium oxidation.

### 3.4. Tribological Properties of the Oxide Scale

**Figure 8:**
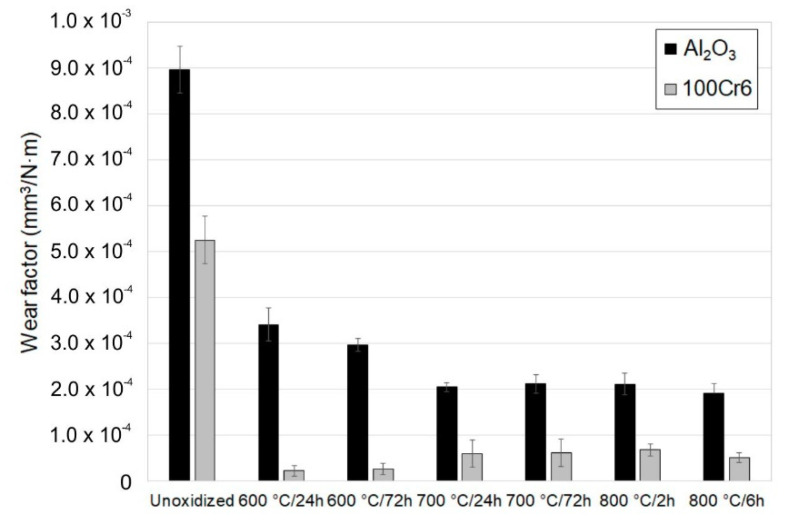
Wear factor of titanium before and after annealing in a ceramic (Al_2_O_3_) and metallic (bearing steel 100Cr6) tribological couple.

**Figure 9:**
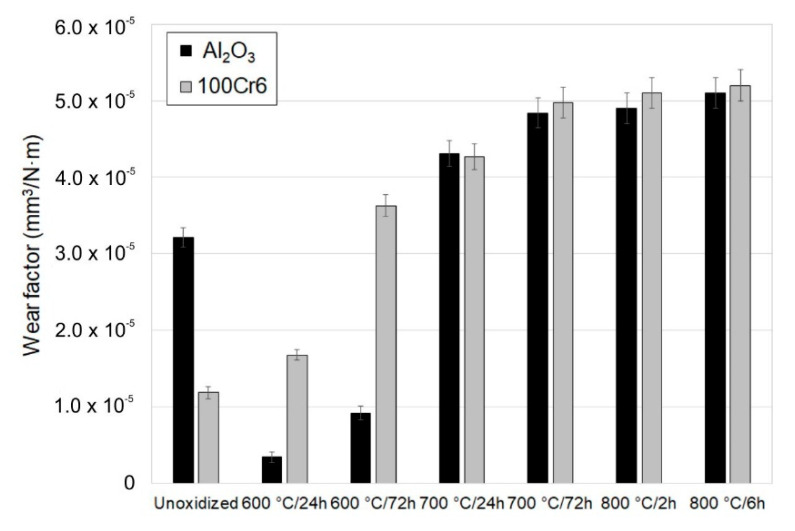
Wear factor of ceramic (Al_2_O_3_) and steel (100Cr6) balls after tribological tests.

**Figure 10:**
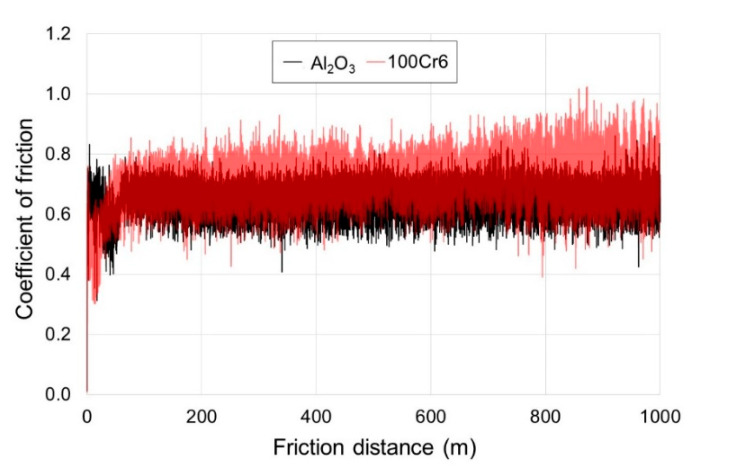
Diagram of the friction coefficient for non-oxidized titanium, recorded during tests with Al_2_O_3_ and 100Cr6 balls.

**Figure 11:**
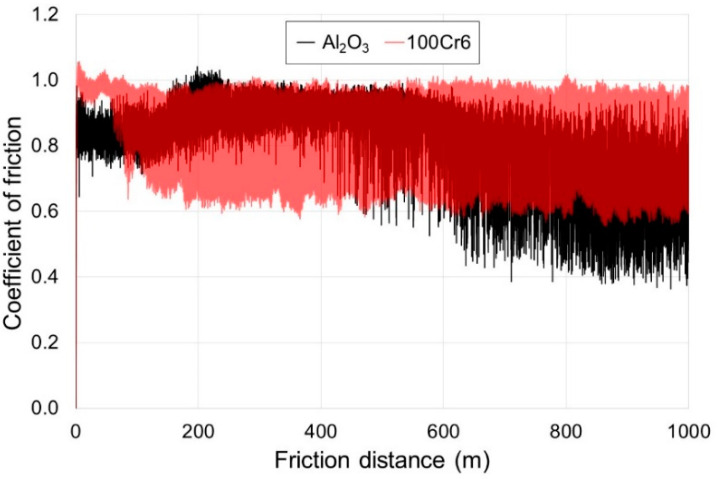
Diagram of the friction coefficient for titanium after annealing at 700 °C (72 h), recorded during friction with Al_2_O_3_ and 100Cr6 balls.

**Figure 12:**
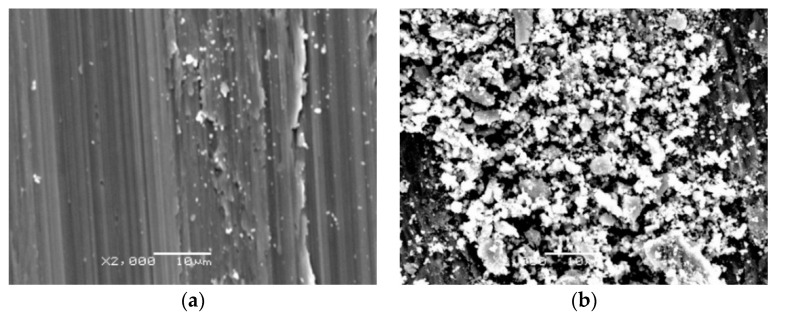
Microscopic photographs of the friction path formed on titanium after tests with Al_2_O_3_ balls (corrugation wear: (**a**) light grey zone, (**b**) dark grey zone).

**Figure 13:**
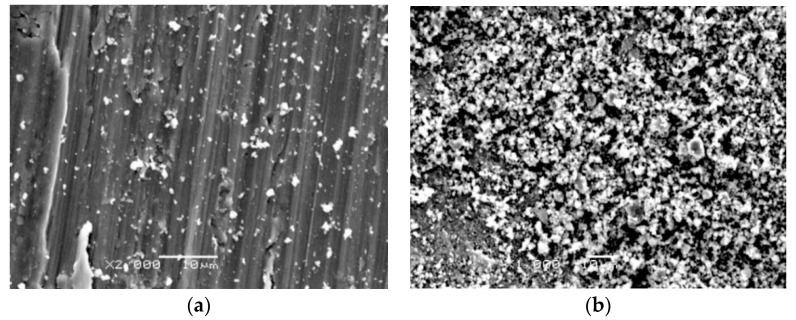
Microscopic photographs of the friction path formed on titanium after tests with 100Cr6 balls (corrugation wear: (**a**) light grey zone, (**b**) dark grey zone).

**Figure 14:**
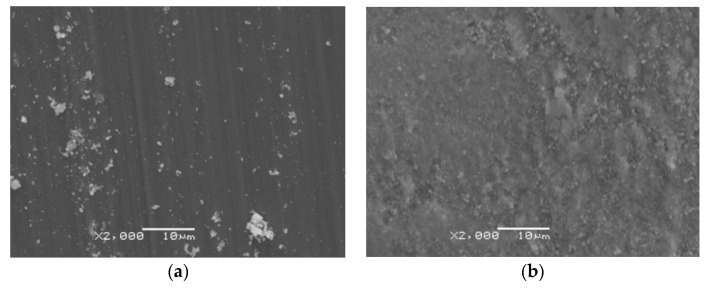
Microscopic photographs of the friction path formed on titanium after annealing at 700 °C for 72 h, after tests with (**a**) Al_2_O_3_ and (**b**) 100Cr6 balls.

Figure 8, Figure 9, Figure 10, Figure 11, Figure 12, Figure 13 and Figure 14 present the results of the tribological tests of titanium before and after thermal treatment at temperatures of 600, 700 (24 h, 72 h) and 800 °C (2 h, 6 h).

The wear factor of titanium was conditioned by the parameters of thermal treatment and the used tribological couple (Al_2_O_3_ and 100Cr6 balls). The highest values of the wear factor were reached for titanium Grade 2 in the non-oxidized condition (Figure 8). At the same time, it was shown that the wear factor in the titanium/Al_2_O_3_ friction couple was approximately 40% higher compared with the titanium/100Cr6 bearing steel couple. The reason for the higher intensity of wear of the disc during friction with ceramic balls was the higher hardness of the Al_2_O_3_ balls. A considerable improvement in the tribological characteristics of the titanium disc was found after oxidation for both the friction couples used. However, a higher reduction in wear factor was found after tribological tests conducted with the 100Cr6 steel balls. This results from the fact that the bearing steel had lower hardness compared with the Al_2_O_3_ balls, which translated directly into lower values of wear factor in the oxidized titanium/steel 100Cr6 friction couple. Furthermore, it was observed that the surface of the friction contact got oxidized during tests with the 100Cr6 balls (Figure 14b), which could also have an additional effect on reducing the wear processes of the titanium Grade 2 disc. During the interaction with the Al_2_O_3_ balls, the lowest value of wear factor was obtained after oxidation of titanium Grade 2 at a temperature of 800 °C for 2 and 6 h. In turn, for the oxidized titanium/steel 100Cr6 friction couple, a slightly lower wear value was found at 600 °C. The reduction in wear factor after isothermal oxidation can be explained by the fact that when two surfaces come into contact with each other, the stress between them causes the surfaces to deform elastically and plastically. Isothermal oxidation leads to a reduction in this phenomenon, which has a beneficial effect on a considerable reduction in friction and wear. Another factor affecting the reduction in sliding wear is a reduced oxygen content in the oxide scale, which has a beneficial effect on the improvement of tribological properties [27,28]. According to Archard’s law, wear factor is inversely proportional to the material hardness [29]. This means that the higher the hardness of the material, the lower the wear. An earlier study by the authors of [30] showed that oxides obtained on titanium were characterized by high hardness, which directly translated into an increased resistance to wear.

Analysis of the wear of ceramic and steel balls leads to the conclusion that its intensity depended on the parameters of the oxidation. It was found that the wear factor of titanium in the non-oxidized condition was more than twice as high for the Al_2_O_3_ ceramic balls. The reason for the higher intensity of wear of the Al_2_O_3_ balls during tests with the non-oxidized surface of titanium Grade 2 may have been the “grain pull-out” mechanism [31]. In addition, the lower resistance to wear of the Al_2_O_3_ balls compared with the 100Cr6 bearing steel balls may have resulted from the higher hardness of the ceramic balls, which in turn, determined the higher wear intensity in the analyzed friction pair. As a result, the tribological interaction between titanium and Al_2_O_3_ balls took place over a larger friction surface, which resulted in higher wear. The lowest wear intensity of the Al_2_O_3_ balls was found after oxidation at 600 °C (especially after 24 h oxidation). After oxidation at 700 and 800 °C, a significant increase was observed in the intensity of wear of the Al_2_O_3_ balls, which could be related to the increase in surface roughness of the oxide layers after annealing. A similar trend in the increase in wear intensity was observed for the 100Cr6 bearing steel balls.

Figure 10 and Figure 11 present examples of the results of measurements of the friction coefficient, obtained during tests using the TRN tribometer. It was demonstrated that during friction of a non-oxidized disc made of titanium with Al_2_O_3_ balls, the mean friction coefficient was ca. 0.65. For the 100Cr6 steel balls, the friction coefficient was slightly higher, and reached 0.71. Moreover, in the tribological test with the 100Cr6 steel balls, a broader amplitude was observed. It was also shown that the friction coefficient for the non-oxidized surface of titanium was lower compared with the oxidized surface. The lower coefficient of friction on the non-oxidized surface could be due to the fact that when a tribological test begins, the roughness of a non-oxidized surface is significantly lower than that of oxidized samples. In addition, there is a thin natural film of TiO_2_ which, in the initial test phase, causes the occurrence of a low friction coefficient [27]. In paper [32], a more non-uniform course of the friction coefficient, with a wide amplitude, was obtained on titanium.

After the annealing of titanium at a temperature of 700 °C, an increase was found in the friction coefficient value, both with the Al_2_O_3_ and the 100Cr6 balls. This is contrary to some literature data [28,32,33,34] which report that oxide layers obtained on titanium and its alloys reduce the value and amplitude of the friction coefficient. The mean friction coefficient after oxidation at 700 °C was ca. 0.82, both for the Al_2_O_3_ and for the 100Cr6 balls. Moreover, it was found for the Al_2_O_3_ balls that the amplitude of the friction coefficient was narrower (up to ca. 400 m), and next, it became much wider. It was also found that for the 100Cr6 balls after exceeding a 50 m friction distance, there was a similar trend.

Microphotographs showing the surface of wear tracks are shown in Figure 12, Figure 13 and Figure 14. Macro- and microscopic observations showed that the friction path on the non-oxidized titanium was characterized by variable width and the presence of irregular elevations and depressions. It was found that the cause of this phenomenon was the so-called corrugation wear (Figure 12 and Figure 13). It was shown that on the path of friction, there were alternating zones of light grey and dark grey shades. The tops of the elevations were visible in the form of light grey areas, while the depressions were dark grey areas. In region A (light grey), a small amount of wear debris was observed. Longitudinal scratches arranged in the same direction were also observed (Figure 12a and Figure 13a). In region B, the wear debris formed during friction was accumulated (Figure 12b and Figure 13b). The wear debris trace may be conducive to the plowing of the worn surface, thus increasing the friction coefficient [35].

Microscopic photographs of fragments of the friction path formed on a titanium disc annealed at 700 °C are presented in Figure 14. After isothermal oxidation, no occurrence of corrugation wear was found. Microscopic observations showed that the friction path after tests with Al_2_O_3_ balls had milder scratches compared with the surface before heat treatment. On the examined surface, the presence of wear debris of different sizes and shapes was also observed. However, after tribological tests carried out using 100Cr6 steel balls, it was observed that the friction surface had undergone oxidation. The oxidation effect of the wear trace surface during interaction with the 100Cr6 bearing steel balls was observed for each tested variant.

The research also included calculations of the wear mechanism indicator. It was shown that its value was close to unity, regardless of the annealing process parameters applied. This means that abrasive wear was the dominant mechanism of wear. This is confirmed by the microscopic images of the wear traces shown in Figure 12, Figure 13 and Figure 14.

In addition, during tribological tests, the phenomenon of partial transfer of wear debris from a titanium disc (before and after annealing) to the surface of the Al_2_O_3_ ceramic balls was observed. The studies showed that such a phenomenon did not occur for the 100Cr6 balls. In the particular case of ceramic sliding over metals, fragments of metal adhere to the ceramic to form a transfer film [36].

### 3.5. Surface Roughness Measurement after Oxidation

**Figure 15:**
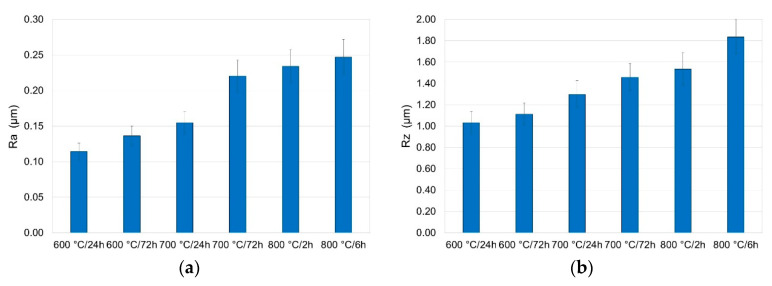
Surface roughness of titanium after annealing at 600, 700 and 800 °C (parameters (**a**) R_a_, (**b**) R_z_).

**Figure 16:**
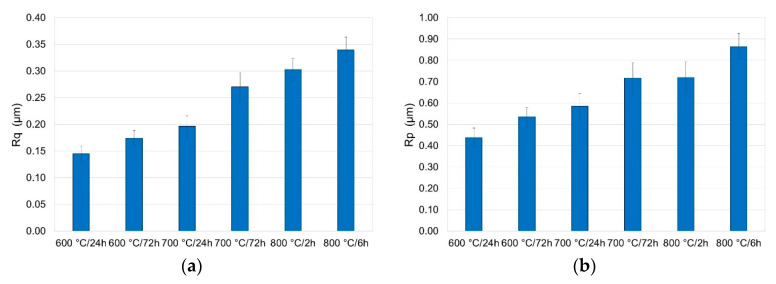
Surface roughness of titanium after annealing at 600, 700 and 800 °C (parameters (**a**) R_q_, (**b**) R_p_).

Roughness measurements made it possible to determine the selected parameters (R_a_, R_z_ R_q_, R_p_) depending on the parameters of oxidation. The results are shown in Figure 15 and Figure 16.

The tests showed that the layers produced had various roughness. It was demonstrated that the surface roughness increased with the increase in temperature. The increase in roughness after oxidation may be due to the formation of large oxide particles on the surface [33]. The lowest value of roughness parameters, i.e., R_a_, R_z_, R_q_ and R_p_, was found for the specimens annealed at a temperature of 600 °C over a period of 6 h. Extending the oxidation time also caused an increase in surface roughness, however, to a lesser extent than the oxidation temperature. The oxides formed at 800 °C over a period of 6 h had the highest roughness.

## 4. Conclusions

The main results and conclusions can be summarized as below:

1. The isothermal oxidation process allowed producing on titanium an oxide scale of good quality, which was characterized by a homogeneous structure and varied thickness depending on the oxidation parameters;
2. The parameters of isothermal oxidation had a significant influence on the course of the oxidation process. It was demonstrated that the oxidation intensity was higher at higher annealing temperatures. This translated directly into the thickness of the oxides produced. The oxide layer obtained after annealing at 800 °C over a period of 6 h had the greatest thickness (8.1 µm);
3. Isothermal oxidation has an adverse effect on the tensile properties of titanium Grade 2. It was found that the maximum reduction in tensile strength was approximately 17.5%, and of the yield point, approximately 13.9%. Isothermal oxidation also had an adverse effect on plastic parameters (reduction in elongation by approximately 12.7%, and reduction of area by approximately 6.1%);
4. Examination of scratch behaviors of the oxide layers by means of a scratch test showed that the layers formed had different adhesion properties. The best scratch resistance was exhibited by the oxide scales obtained at 800 °C, in particular, the layer produced after 6-h oxidation (L_c1_ = 63 N, L_c2_ = 85 N);
5. Titanium after isothermal oxidation was characterized by more advantageous tribological properties. The reason for the significant improvement in resistance to sliding wear was the presence of oxides distinguished by their high hardness. At the same time, it was shown that the oxide scale caused a significant increase in the friction coefficient, which was strictly connected with the increased surface roughness after isothermal oxidation. It was found that the principal wear mechanism was abrasive wear;
6. Roughness measurements showed that an increase in oxidation temperature is a factor which intensely increases surface roughness. It was found that extending the oxidation time also increases surface roughness, but to a lesser extent than the oxidation temperature.

## Figures and Tables

**Table 1:** Critical load values.

Specimen Condition	Critical Load [N]	
	L_c1_	L_c2_
600 °C/24 h	39	75
600 °C/72 h	35	75
700 °C/24 h	34	67
700 °C/72 h	47	81
800 °C/2 h	50	82
800 °C/6 h	63	85

## References

[1] Lederer S., Lutz P., Fürbeth W. (2018). Surface modification of Ti 13Nb 13Zr by plasma electrolytic oxidation. Surf. Coat. Technol..

[2] Hu H.Y., Zhang L., He Z.Y., Jiang Y.H., Tan J. (2019). Microstructure evolution, mechanical properties, and enhanced bioactivity of Ti-13Nb-13Zr based calcium pyrophosphate composites for biomedical applications. Mater. Sci. Eng. C.

[3] Garbacz H., Wieciński P., Kuczyńska D., Kubacka D., Kurzydłowski K.J. (2018). The effect of grain size on the surface properties of titanium grade 2 after different treatments. Surf. Coat. Technol..

[4] Koizumi H., Takeuchi Y., Imai H., Kawai T., Yoneyama T. (2019). Application of titanium and titanium alloys to fixed dental prostheses. J. Prosthodont. Res..

[5] Gravinaa A.N., Rubert A.A., Bertuola M., Fernández Lorenzo de Mele M. (2019). Bioactivity enhancement of cerium-containing titanium oxide nanotubes. Relationship between surface reactivity and nanostructuring process. Surf. Coat. Technol..

[6] Jebieshia T.R., Kim J.M., Kang J.W., Son S.W., Kim H.D. (2020). Microstructural and very high cycle fatigue (vhcf) behavior of Ti_6_Al_4_V—A comparative study. Materials.

[7] Salguero J., Del Sol I., Vazquez-Martinez J.M., Schertzer M.J., Iglesias P. (2019). Effect of laser parameters on the tribological behavior of Ti_6_Al_4_V titanium microtextures under lubricated conditions. Wear.

[8] Redmore E., Li X., Dong H. (2019). Tribological performance of surface engineered low-cost beta titanium alloy. Wear.

[9] Hong X., Tan Y.-F., Wang X.-L., Tan H., Xu T. (2015). Effects of nitrogen flux on microstructure and tribological properties of in-situ TiN coatings deposited on TC11 titanium alloy by electrospark deposition. Trans. Nonferrous Met. Soc. China.

[10] Budinski K.G. (1991). Tribological properties of titanium alloys. Wear.

[11] Miller P.D., Holladay J.W. (1958). Friction and wear properties of titanium. Wear.

[12] An Q., Chen J., Tao Z., Ming W., Chen M. (2020). Experimental investigation on tool wear characteristics of PVD and CVD coatings during face milling of Ti-6242S and Ti-555 titanium alloys. Inter. J. Refrac. Met. Hard Mater..

[13] Acciari H.A., Palma D.P.S., Codaro E.N., Zhou Q., Wang J., Ling Y., Zhang J., Zhang Z. (2019). Surface modifications by both anodic oxidation and ion beam implantation on electropolished titanium substrates. App. Surf. Sci..

[14] Alcázar J.C.B., Lemos R.M.J., Conde M.C.M., Chisini L.A., Salas M.M.S., Noremberg B.S., Da Motta F.V., Demarco F.F., Tarquinio S.B.C., Carreño N.L.V. (2019). Preparation, characterization, and biocompatibility of different metal oxide/PEG-based hybrid coating synthesized by sol–gel dip coating method for surface modification of titanium. Prog. Org. Coat..

[15] Borgioli F., Galvanetto E., Iozzelli F., Pradelli G. (2005). Improvement of wear resistance of Ti–6Al–4V alloy by means of thermal oxidation. Mater. Lett..

[16] Tillmann W., Grisales D., Stangier D., Jebara I.B., Kang H. (2019). Influence of the etching processes on the adhesion of TiAlN coatings deposited by DCMS, HiPIMS and hybrid techniques on heat treated AISI H11. Surf. Coat. Technol..

[17] Zhang X., Tian X.-B., Zhao Z.-W., Gao J.-B., Zhou Y.-W., Gao P., Guo Y.-Y., Lv Z. (2019). Evaluation of the adhesion and failure mechanism of the hard CrN coatings on different substrates. Surf. Coat. Technol..

[18] Aniołek K., Kupka M. (2019). Mechanical, tribological and adhesive properties of oxide layers obtained on the surface of the Ti–6Al–7Nb alloy in the thermal oxidation process. Wear.

[19] Aniołek K., Kupka M., Barylski A., Dercz G. (2015). Mechanical and tribological properties of oxide layers obtained on titanium in the thermal oxidation process. App. Surf. Sci..

[20] Camarano A., Giuranno D., Narciso J. (2020). SiC-IrSi_3_ for high oxidation resistance. Materials.

[21] Bailey R., Sun Y. (2013). Unlubricated sliding friction and wear characteristics of thermally oxidized commercially pure titanium. Wear.

[22] Biswas A., Majumdar J.D. (2009). Surface characterization and mechanical property evaluation of thermally oxidized Ti–6Al–4V. Mater. Character..

[23] Dalili N., Edrisy A., Farokhzadeh K., Li J., Lo J., Riahi A.R. (2010). Improving the wear resistance of Ti–6Al–4V/TiC composites through thermal oxidation (TO). Wear.

[24] Aniołek K., Barylski A., Kupka M. (2018). Modelling the structure and mechanical properties of oxide layers obtained on biomedical Ti-6Al-7Nb alloy in the thermal oxidation process. Vacuum.

[25] Jiang H., Hirohasi M., Lu Y., Imanari H. (2020). Effect of Nb on the high temperature oxidation of Ti–(0–50 at.%) Al. Scr. Mater..

[26] Abduluyahed A.A., Kurzydłowski K.J. (1998). Tensile properties of a type 316 stainless steel strained in air and vacuum. Mater. Sci. Eng. A.

[27] Wang S., Liao Z., Liu Y., Liu W. (2014). Influence of thermal oxidation temperature on the microstructural and tribological behavior of Ti6Al4V alloy. Surf. Coat. Technol..

[28] Arslan E., Totik Y., Demirci E., Alsaran A. (2010). Influence of surface roughness on corrosion and tribological behavior of CP-Ti after thermal oxidation treatment. J. Mater. Eng. Perform..

[29] Fellah M., Assala O., Labaïz M., Dekhil L., Iost A. (2013). Friction and wear behavior of Ti-6Al-7Nb biomaterial alloy. J. Biomater. Nanobiotech.

[30] Aniołek K., Kupka M., Barylski A., Mieszczak Ł. (2016). Characteristic of oxide layers obtained on titanium in the process of thermal oxidation. Arch. Metall. Mater..

[31] Guleryuz H., Cimenoglu H. (2005). Surface modification of a Ti–6Al–4V alloy by thermal oxidation. Surf. Coat. Technol..

[32] Siva Rama Krishna D., Brama Y.L., Sun Y. (2007). Thick rutile layer on titanium for tribological applications. Tribol. Int..

[33] Wang S., Liao Z., Liu Y., Liu W. (2015). Influence of thermal oxidation duration on the microstructure and fretting wear behavior of Ti_6_Al_4_V alloy. Mater. Chem. Phys..

[34] Dearnley P.A., Dahm K.L., Çimenoglu H. (2004). The corrosion–wear behaviour of thermally oxidised CP-Ti and Ti–6Al–4V. Wear.

[35] Duarte M., Vragovic I., Molina J.M., Prieto R., Narciso J., Louis E. (2009). 1/*f* Noise in Sliding Friction under Wear Conditions: The Role of Debris. Phys. Rev. Lett..

[36] Duarte M., Molina J.M., Prieto R., Louis E., Narciso J. (2007). Self-similar fluctuations and 1/*f* noise in dry friction dynamics. Metall. Mater. Trans. A.

